# Plasmid-based and -free methods using CRISPR/Cas9 system for replacement of targeted genes in *Colletotrichum sansevieriae*

**DOI:** 10.1038/s41598-019-55302-8

**Published:** 2019-12-12

**Authors:** Masayuki Nakamura, Yuta Okamura, Hisashi Iwai

**Affiliations:** Faculty of Agriculture, Kagoshima University, Kagoshima, Japan

**Keywords:** Fungal genetics, Fungal genetics

## Abstract

The CRISPR-Cas9 system has a potential for wide application in organisms that particularly present low homologous integration rates. In this study, we developed three different methods using this system to replace a gene through homology-directed repair in the plant pathogenic fungus *Colletotrichum sansevieriae*, which has a low recombination frequency. The gene encoding scytalone dehydratase was used as the target so that mutants can be readily distinguished owning to a lack of melanin biosynthesis. First, we performed a plasmid-based method using plasmids containing a Cas9 expression cassette and/or a single-guide RNA (sgRNA) under the control of the endogenous U6 snRNA promoter, and 67 out of 69 (97.1%) transformants exhibited a melanin-deficient phenotype with high efficiency. Second, we performed a transformation using a Cas9 protein/sgRNA complex and obtained 23 out of 28 (82.1%) transformants. Lastly, we developed a hybrid system combining a Cas9 protein and donor DNA-sgRNA expression plasmid, which yielded 75 out of 84 (89.2%) transformants. This system was also applicable to four other genes at different loci of the fungus. This is the first study to establish a CRISPR/Cas9 gene replacement system in *Colletotrichum* spp. and it presents a potential application for a broad range of use in other species of the genus.

## Introduction

*Colletotrichum sansevieriae* is an ascomycete fungus causing anthracnose disease only in plants of the genus *Sansevieria*, indicating its high host specificity^1^. The draft genome analysis of the fungus has been conducted, and its effector candidates and other pathogenicity-related factors have been predicted^2^. Thus, developing an efficient and convenient approach to clarify the important factors for pathogenicity of the fungus has become critical. To date, the generation of mutants of *C. sansevieriae* has been performed through spontaneous homologous recombination and *Agrobacterium tumefaciens*-mediated transformation^3^. However, these techniques are insufficient and time-consuming for screening target mutants.

The CRISPR/Cas9 system is the bacterial and archaeal adaptive immune system^4,5^, which has already been used to disrupt or replace genes in various organisms such as yeasts, plants, fish, animals, and filamentous fungi^6–10^. In plant pathogenic fungi, the establishment of the system has been reported in *Fusarium oxysporum*^11^, *Magnaporthe oryzae*^12–14^, *Phytophthora* spp.^15–17^, *Sclerotinia sclerotiorum*^18^ and *Ustilago* spp.^19,20^ to date. However, the system is yet to be established in *Colletotrichum* spp., which are devastating pathogens that are agriculturally and economically relevant. The CRISPR/Cas9 system comprises the following two principal components for gene targeting and cleavage: a Cas9 endonuclease and a single-guide RNA (sgRNA)^21^. The sgRNA contains a specific RNA sequence (20 nucleotides) that recognizes the target DNA followed by the protospacer-adjacent motif (PAM) and guides the Cas9 there for editing, leading to the introduction of a double-strand DNA break (DSB) at the defined locus^22^. The DSB can be repaired by non-homologous end-joining (NHEJ) or homology-directed repair (HDR)^23–25^. The NHEJ pathway can create uncontrollable mutations (indels), resulting in the loss-of-function of a target gene. Contrastingly, the HDR pathway is a more accurate mechanism for DSB repair because it requires donor DNA homologous to the sequences flanking the DSB. In this study, for precise genome editing, we mainly focus on a HDR-based knock-in strategy.

Here, we established the following three different methods using the CRISPR/Cas9 system in *C. sansevieriae* to replace a specific gene via the HDR pathway: (i) the plasmid-based system, (ii) the plasmid-free system (Cas9 nuclease/sgRNA complexes), and (iii) the hybrid system. For the plasmid-based strategy, the endogenous U6 snRNA promoter in *C. sansevieriae* was used to express sgRNA. For the hybrid system, a combination of a Cas9 protein and a donor DNA-sgRNA expression vector was employed. As a target gene, the scytalone dehydratase gene (*SCD1*) was targeted to replace so that mutants can be easily screened based on the loss of the ability to synthesize melanin. To the best of our knowledge, this is the first study to report the use of a CRISPR/Cas9 system in *Colletotrichum* spp., which provides an efficient and useful strategy for finding the functions of specific genes involved in pathogenicity in the species of the genus.

## Results

### Identification of RNA polymerase III-based U6 promoters

**Figure 1:**
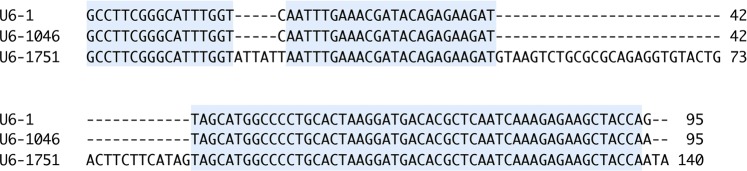
Alignment of the U6 snRNA genes of *Aspergillus fumigatus* (U6-1^28^) and *Colletotrichum sansevieriae*. Two snRNAs were identified from the database of *C. sansevieriae* (NJHP000000002) and were designated as U6-1046 and U6-1751, respectively. Identical sequences are highlighted.

To identify RNA polymerase III promoters in the genome of *C. sansevieriae*, we searched its draft genome (NJHP00000000^2^) for U6 snRNA genes which are highly conserved from microbes to mammals^26^. Two candidate genes were found in the genome and we designated them as U6-1046 and U6-1751, respectively, according to the contig numbers in which the genes were located. The two genes have high homology with the U6 snRNA^27,28^ from *Aspergillus fumigatus* (Fig. 1). However, U6-1751 contains extra regions which are not found in the other two genes. Therefore, to express sgRNA, we used the 500-bp upstream promoter (P_U6-1046_) and 500-bp downstream terminator (T_U6-1046_) regions of U6-1046 that share similarity (98.9% identity) to the sequence of the U6 snRNA of *A. fumigatus*.

### Gene replacements with conventional knock-in and plasmid-based CRISPR/Cas9

**Figure 2:**
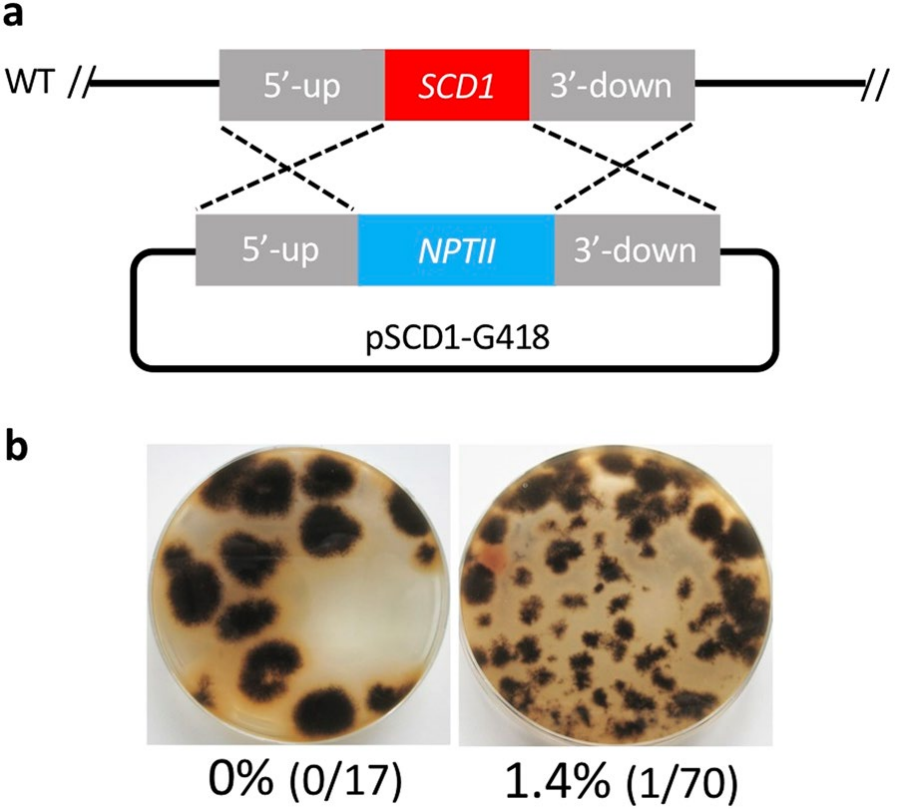
The conventional method of gene replacement in *Colletotrichum sansevieriae*. (**a**) Knock-in of the *SCD1* locus by the donor DNA, based on *NTPII* as a selectable marker. WT, wild type. (**b**) Plates from transfecting pSCD1-G418 into protoplasts at concentrations of 5 × 10^6^ (left) and 1 × 10^8^ (right). Percentage of pink colonies with a melanin-deficient phenotype are shown under the plate images.

To compare the efficiency of gene replacements by the conventional method and CRISPR/Cas9 via the HDR pathway, we first used only the replacement plasmid (pSCD1-G418) containing the marker gene, *NPTII*, which is inserted between the flanking homologous regions of the target gene, *SCD1*, which is involved in melanin biosynthesis (Fig. 2a). Two different concentrations of protoplasts were subjected to polyethylene glycol (PEG)-mediated chemical transformation^29^. In a 5 × 10^6^-protoplast assay, not a single melanin-deficient colony (colored in pink) was obtained; moreover, even in a 1 × 10^8^-protoplast assay, only one pink colony out of 70 was obtained, indicating a 1.4% transformation efficiency (Fig. 2b).

**Figure 3:**
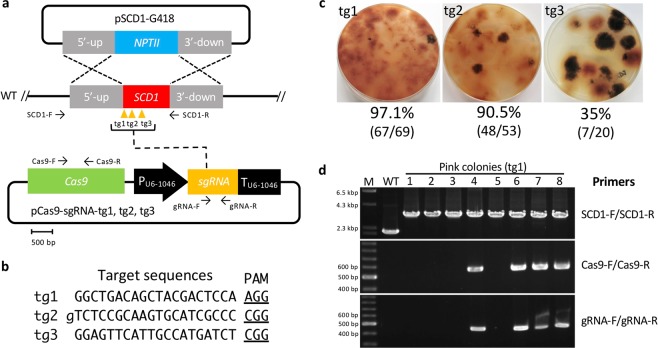
Plasmid-based CRISPR/Cas9 with donor-DNA and Cas9-sgRNA expression plasmids. (**a**) Gene replacement of the *SCD1* locus via the homology-directed repair pathway. Arrows represent the primers used. WT, wild type (**b**) Three target and PAM sequences (tg1, tg2, and tg3) designed for single guide RNAs are shown. Guanine (lower-case initial) in tg2 is added to enable efficient expression from the U6 promoter (P_U6-1046_). (**c**) Pink colonies generated using each target site and the percentage of pink-colony emergence. (**d**) Confirmation of *SCD1* replacement (upper), Cas9-cassette integration (middle) and sgRNA-cassette integration (bottom) by PCR. Primers used are shown on the right. M, DNA marker; WT, wild type; lanes 1–8, pink colonies generated with tg1. The full-length gels are shown in Fig. S1.

**Figure 4:**
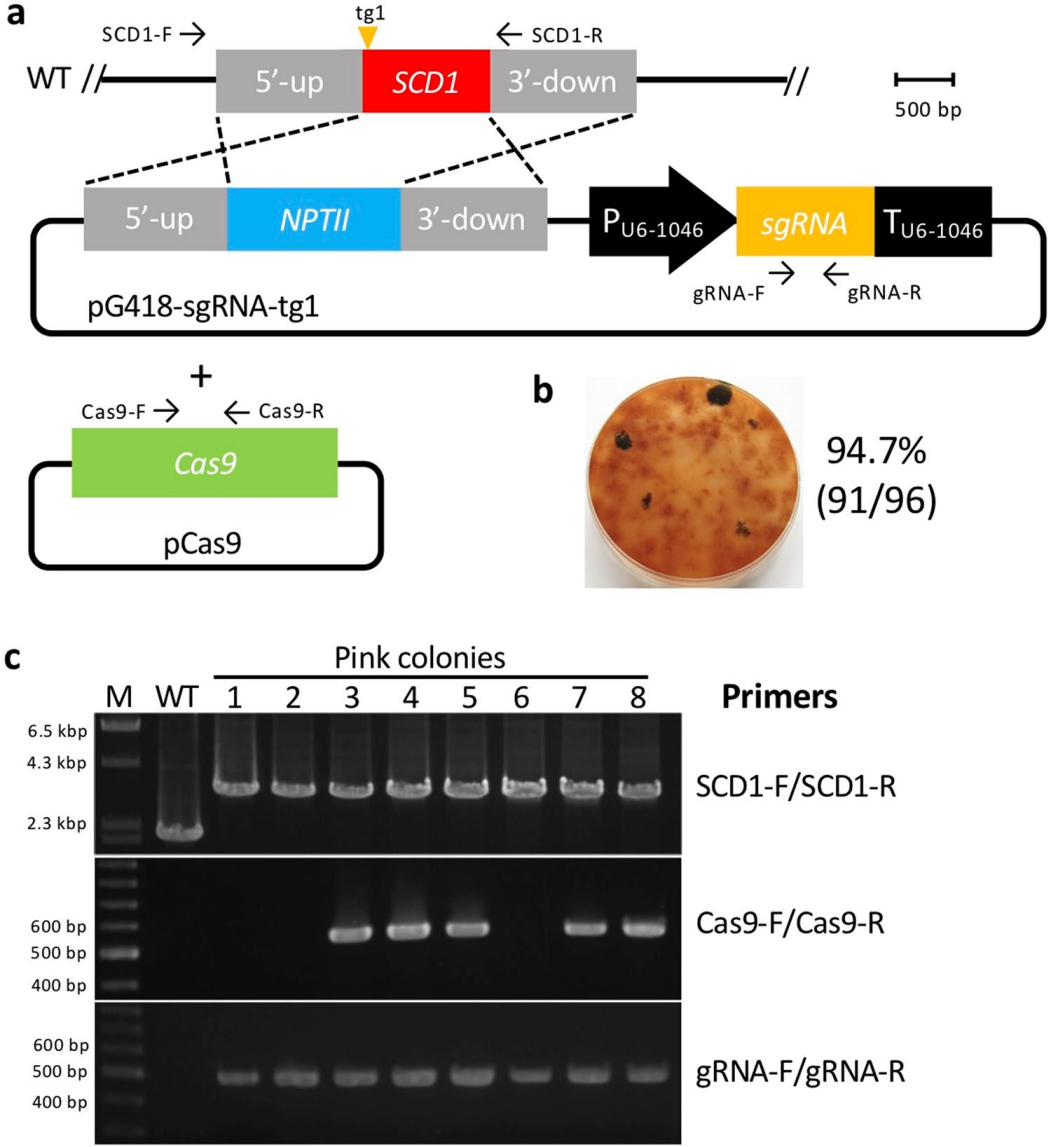
Plasmid-based CRISPR/Cas9 with Cas9 and donor DNA-sgRNA expression plasmids. (**a**) Gene replacement of the *SCD1* locus via the homology-directed repair pathway. Arrows represent primers used. WT, wild type. (**b**) Pink colonies generated with tg1 and the percentage of pink-colony emergence. (**c**) Confirmation of *SCD1* replacement (upper), Cas9-cassette integration (middle) and sgRNA-cassette integration (bottom) by PCR. Primers used are shown on the right. M, DNA marker; WT, wild type; lanes 1–8, pink colonies. The full-length gels are shown in Fig. S2.

We next performed a CRISPR/Cas9 technique using a plasmid containing both Cas9 and sgRNA expression cassettes together with pSCD1-G418 (Fig. 3a). We employed a *Cas9* gene that was codon-optimized for the filamentous fungus *A. niger*. The *Cas9* gene with a SV40 nuclear localization signal (NLS) and under the control of the *tef1* promoter and terminator of *A. nidulans* was amplified from pFC332^30^ and cloned into expression plasmids. The following three different target sites were designed on the *SCD1* locus: target site 1 (tg1), tg2, and tg3 (Fig. 3b); tg1 and tg3 start with guanine; however, tg2 starts with thymine. Therefore, we added extra guanine at the 5′-end of tg2 to enable efficient expression from the U6 promoter. For further experiments, a concentration of 5 × 10^6^ protoplasts per assay was employed. When the plasmid (pCas9-sgRNA-tg1) harboring the sgRNA of tg1 was used, 67 out of the 69 (97.1%) colonies were pink, giving the highest efficiency; meanwhile, 48 out of 53 (90.5%) colonies were obtained for tg2, and 7 out of 20 (35%) colonies were obtained for tg3 (Fig. 3c), indicating that the replacement efficiency was dependent on the target site. Eight pink colonies created using pCas9-sgRNA-tg1 were randomly selected and analyzed by PCR. All the transformants exhibited the correct gene replacement via double crossover (Fig. 3d); longer fragments are supposed to be amplified using the primer set SCD1-F/SCD1-R (Table S1, Fig. 3a) from the target locus rather than the wild type when the correct gene replacement occurs. Four out of the 8 transformants did not harbor the Cas9-sgRNA expression cassettes. We also performed a co-transformation using the Cas9 expression plasmid (pCas9) and the donor DNA-sgRNA expression plasmid (pG418-sgRNA-tg1) (Fig. 4a). Ninety-one pink colonies out of 96 (94.7%) were obtained, indicating a high efficiency (Fig. 4b). With 8 pink colonies randomly chosen, all exhibited precise gene replacement events (Fig. 4c). Although the sgRNA cassette was integrated into all the transformants, three of them did not acquire the *Cas9* gene. The above results clearly demonstrate that the CRISPR/Cas9 system is much more efficient than the conventional method to replace a precise gene via the HDR pathway in *C. sansevieriae*.

### Influence of the length of flanking homologous regions on the efficiency of CRISPR/Cas9

**Figure 5:**
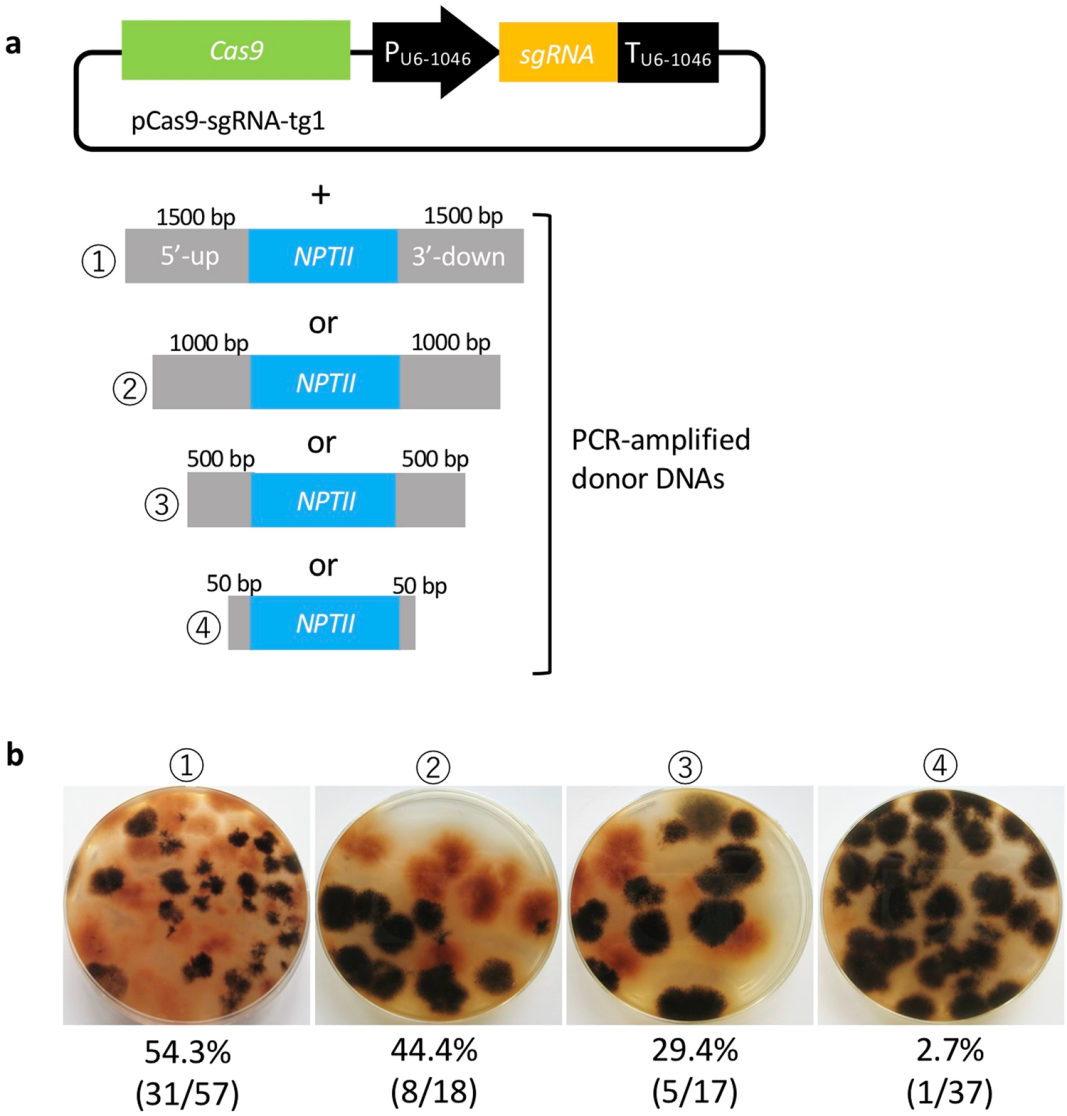
Influence of the length of flanking homologous regions in donor DNA on gene replacement events via CRISPR/Cas9. (**a**) Plasmid containing Cas9-sgRNA (tg1) expression cassettes and PCR-amplified donor DNAs were co-transformed. (**b**) Percentage of pink-colony emergence in each different length of the flanking regions. The numbers (1–4) correspond to the numbers of the donor DNAs.

We investigated the effect of the length of flanking homologous regions on the replacement frequency of the CRISPR/Cas9 system. Arm lengths flanking the *SCD1* gene ranged in size from 50 to 1500 bp. Donor DNAs containing different lengths of the flanking regions on each side of the selectable marker (*NPTII*) were prepared using PCR amplification using pSCD1-G418 as a template. The donor DNAs were co-transfected with pCas9-sgRNA-tg1 (Fig. 5a). Consequently, the emergence of melanin-deficient colonies occurred at frequencies of 54.3% (31/57), 44.4% (8/18), 29.4% (5/47), and 2.7% (1/37) when using donor DNA carrying flanking regions of 1500 bp, 1000 bp, 500 bp and 50 bp, respectively (Fig. 5b). These results indicate that longer flanking regions result in more efficient homologous recombination.

### Gene replacement using *in vitro*-assembled Cas9/sgRNA complex

**Figure 6:**
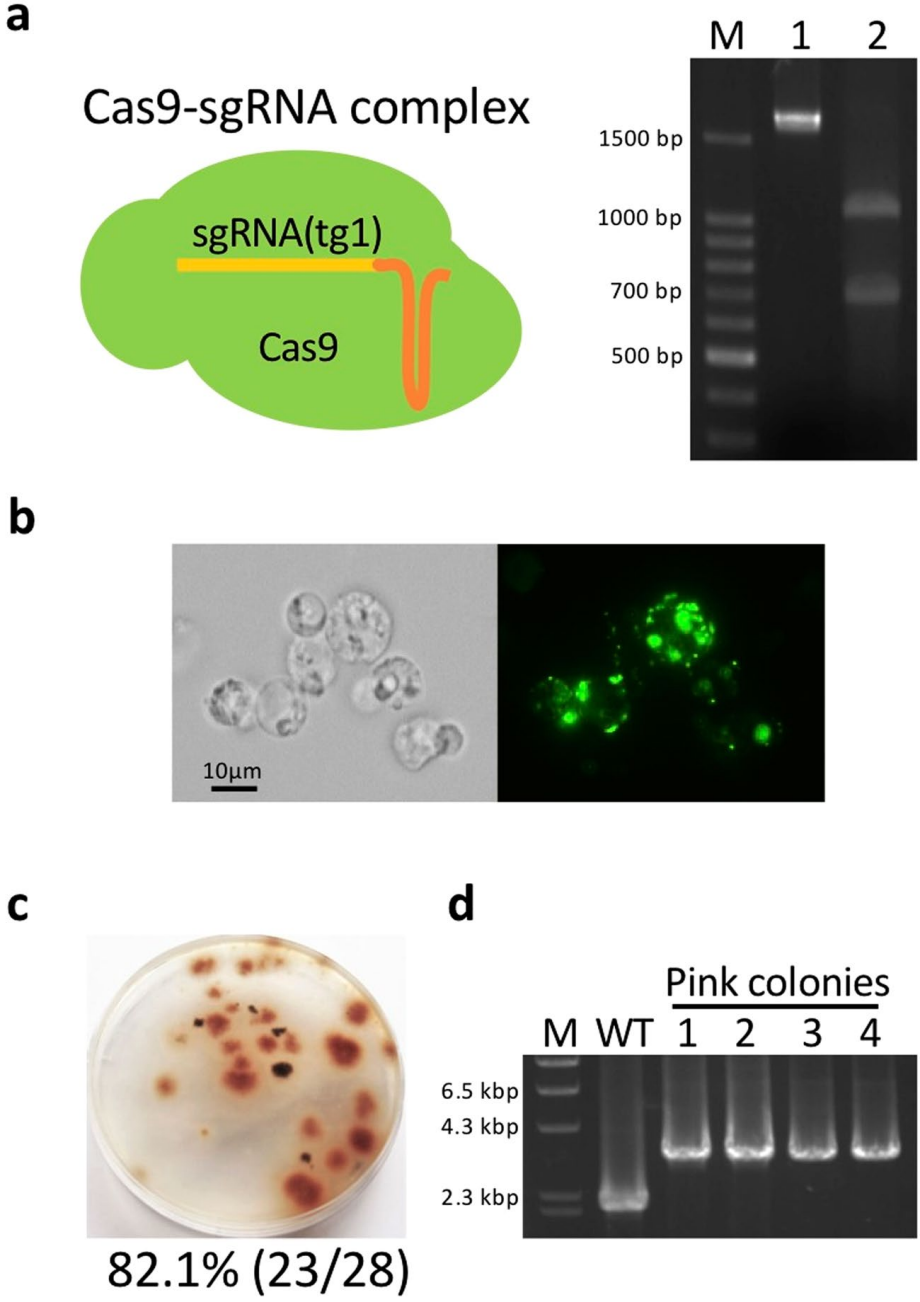
Transformation by *in vitro*-assembled Cas9/sgRNA complex. (**a**) Digestion of *SCD1* fragments without the Cas9/sgRNA complex (lane 1) or with the complex (lane 2). M, DNA marker. The full-length gel is shown in Fig. S3. (**b**)Transfection of Cas9 protein fused with GFP into protoplasts via polyethylene glycol-mediated chemical transformation. (**c**) Percentage of pink-colony emergence. (**d**) Confirmation of *SCD1* replacement by PCR. M, DNA marker; WT, wild type; lanes 1–4, pink colonies. The full-length gel is shown in Fig. S4.

We also developed a plasmid-free genome editing system. Commercial Cas9 nucleases and *in vitro*-transcribed sgRNAs were assembled, yielding a Cas9/sgRNA complex. To check whether the complex can recognize and cleave the target site (tg1), it was incubated with 1716-bp PCR fragments containing the *SCD1* gene; consequently, two digested fragments with expected sizes of 688 bp and 1028 bp were generated (Fig. 6a). Next, to test whether Cas9 nucleases can be transfected into protoplasts through PEG-mediated transformation, Cas9 proteins fused with GFP were used. GFP fluorescence was clearly observed in transfected protoplasts (Fig. 6b), indicating that Cas9 nucleases can be subjected to PEG-mediated transformation. Using this method, pink colonies emerged at a frequency of 82.1% (23/28) (Fig. 6c) and four transformants randomly chosen all indicated correct gene replacement (Fig. 6d), demonstrating that the Cas9/sgRNA complex system also works well in *C. saensevieriae* through PEG-mediated transformation.

### Utilization of the hybrid system with a Cas9 protein and a sgRNA expression plasmid

**Figure 7:**
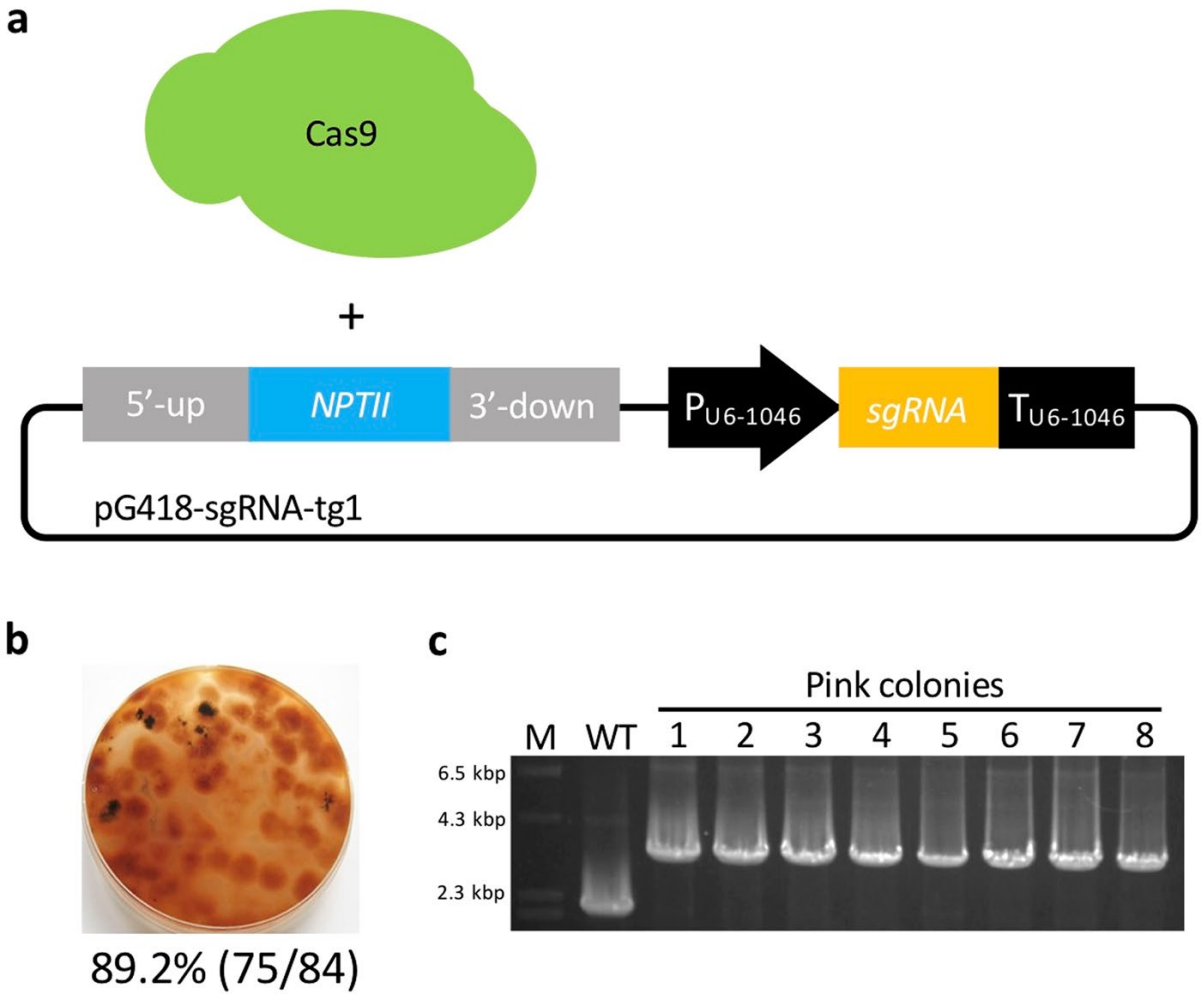
The hybrid system of CRISPR/Cas9. (**a**) The combination of Cas9 protein and donor DNA-sgRNA (tg1) expression plasmid. (**b**) Percentage of pink-colony emergence. (**c**) Confirmation of *SCD1* replacement by PCR. M, DNA marker; WT, wild type; lanes 1–8, pink colonies. The full-length gel is shown in Fig. S5.

**Figure 8:**
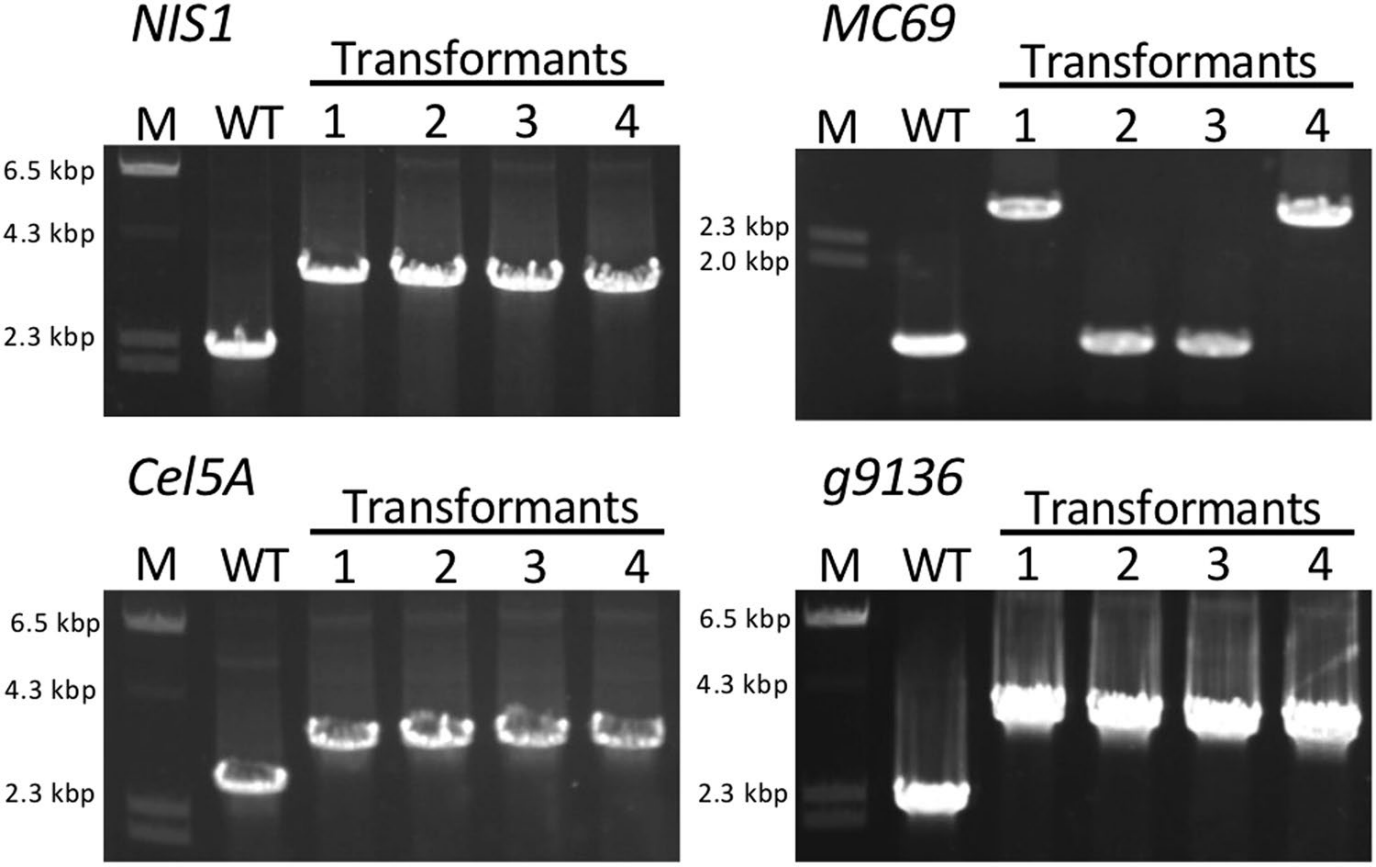
Application of the hybrid system to four additional genes at the different genomic loci of *Colletotrichum sansevieriae*. PCR analyses of replacement events for each gene are shown. *NIS1* and *MC69*, effector genes; *Cel5A*, a cellulase gene; *g9136*, a gene of unknow function. M, DNA marker; WT, wild type; lanes 1–4, transformants. The full-length gels are shown in Fig. S6.

We wondered whether the combination of a Cas9 protein and a plasmid containing donor DNA-sgRNA expression cassettes would work successfully, so we attempted to replace *SCD1* using Cas9 protein and pG418-sgRNA-tg1 (Fig. 7a). Consequently, pink colonies were obtained at a high frequency of 89.2% (75/84) (Fig. 7b). Eight randomly chosen transformants showed correct gene replacement events (Fig. 7c), indicating that this hybrid system is a viable alternative strategy to edit genomes. To further investigate whether other genomic loci can be similarly replaced using this hybrid system, two additional effector genes (*NIS1* and *MC69*), a cellulase gene (*Cel5A*), and a gene of unknown function (*g9136*) were targeted in *C. sansevieriae*. The G418-resistant colonies obtained ranged from 30 to 60 in each transformation, and four transformants were randomly selected from each assay to confirm the occurrence of gene replacements by PCR (Fig. 8). All four transformants from the assays for *NIS1*, *Cel5A*, and *g9136*, and two from that for *MC69* exhibited the precise gene replacement events, demonstrating that this hybrid system is also applicable to other loci in *C. sansenvieriae*.

## Discussion

In this study, we developed three different methods using the CRISPR/Cas9 system for precise gene replacements in *C. sansevieriae*. To the best of our knowledge, this is the first study to establish a CRISPR/Cas9 gene replacement system in *Colletotrichum* spp. Using the conventional method, the replacement frequencies of the *SCD1* gene were 0% and 1.4% when 5 × 10^6^ and 1 × 10^8^ protoplasts, respectively, were used (Fig. 2). However, the established plasmid-based CRISPR/Cas9 caused the desired gene replacement at high frequencies of 97.1% (Figs. 3c) and 94.7% (Fig. 4b) in 5 × 10^6^-protoplast assays, clearly indicating that the CRISPR/Cas9 system is an efficient and useful technique to replace a gene in *C. sansevieriae*, which has a low recombination frequency. In addition, we investigated the influence of the length of flanking homologous regions (50 to 1500 bp) on the efficiency of double crossover via CRISPR/Cas9. Longer flanking regions resulted in more melanin-deficient colonies (Fig. 5). da Silva Ferreira *et al*.^31^ also reported that longer flanking regions (1500 to 2000 bp) increased the frequency of homologous integration, and that flanking regions shorter than 500 bp did not yield any homologous integration in *A. fumigatus*. In contrast to this report, Zhang *et al*.^28^ reported that only a 39-bp arm is enough to repair the DNA breaks created by the CRISPR/Cas9 system in *A. fumigatus*. This research group designed the short arm to contain the sequences proximally located to the PAM site, which may favorably lead to the induction of microhomology-mediated end joining. The reason why the frequency of homologous recombination and the total number of G418-resistant colonies in *C. sansevieriae* were lower when PCR-amplified donor DNAs were used instead of plasmids is probably that the donor fragments, to some degree, might have been degraded in fungal cells and/or preferentially ectopically integrated into the genome because they were linear. Therefore, as a donor DNA, circular plasmids may be suitable to replace a precise gene without ectopic integration. We also performed a transformation involving the NHEJ repair pathway; however, only a few pink colonies were recovered (data not shown). Interestingly all the transformants acquired large insertions at the target sites, although repair of cleavage via NHEJ usually creates small indels^32–34^. Li *et al*.^18^ also reported that all target sites in *S. sclerotiorum* harbored large insertions that were generated by the rearrangement of plasmid segments in a cell. Genome editing by the NHEJ repair pathway may not be suitable for *C. sansevieriae*.

We next tested the direct transfection of an *in-vitro* assembled Cas9/sgRNA complex into *C. sansevieriae* via PEG-mediated transformation. Cas9 proteins were successfully transfected into protoplasts (Fig. 6b). Although we obtained less G418-resistant colonies in total than that from the plasmid-based transformation, pink colonies emerged at a high frequency of 82.1% (Fig. 6c). This technique is useful especially when endogenous RNA polymerase III promoters are unknown.

In the current study, we also attempted a hybrid transformation using a Cas9 protein and a sgRNA expression plasmid simultaneously (Fig. 7a). Contrary to our expectations, we achieved much better results. We obtained pink colonies at a high frequency of 89.2% (Fig. 7b). All randomly selected colonies showed the correct gene replacements (Fig. 7c), indicating that this hybrid system is an alternative technique using the CRISPR/Cas9 system. Furthermore, to investigate whether this hybrid system is applicable to other genomic loci of *C. sansevieriae*, four additional genes (*NIS1, MC96, Cel5A*, and *g9136*) were also targeted. All transformants randomly chosen from the replacement assays for *NIS1, Cel5A*, and *g9136* exhibited precise gene replacements, while half of those for *MC69* exhibited correct gene replacements (Fig. 8). The replacement frequency of *MC69* was 50%, which is notably much higher than that by the conventional method because we designed only one target site in this gene. It has been speculated that the activity of the Cas9 nuclease depends on target sites, and similar to previous findings^12,35^, in the present study, one (tg3) of the three target sites in *SCD1* did not function appropriately (Fig. 3c). Targeted transformants obtained from the hybrid system can be used immediately for a subsequent gene complementation assay without confirming the integration of plasmids because a Cas9 protein is used directly instead of transfecting its gene via a plasmid vector. It has been reported that the constitutive expression of Cas9 increases the number of off-target mutations and triggers DNA damage responses^36,37^. However, the hybrid system and the system with Cas9/sgRNA complexes can avoid this problem. Moreover, kits for *in vitro* transcription of sgRNAs are expensive. Since gene disruption/replacement techniques are routine laboratory methods, it is desirable for them to be inexpensive. Based on our findings, we may propose that the hybrid system is a convenient, economical, and versatile technique not only for *Colletotrichum* spp., but also for many different kinds of organisms.

## Materials and Methods

### Strains and growth conditions

*C. sansevieriae* Sa-1-21 was grown on potato dextrose agar (PDA) or potato dextrose broth (PDB) (Becton Dickinson, Sparks, USA) at 25 °C. *Escherichia coli* JM109 (Takara Bio, Otsu, Japan) was grown at 37 °C in Luria-Bertani broth for all routine purposes. Antibiotics were added to the media at the following concentrations: 100 µg/mL ampicillin for *E. coli*, 400 µg/mL G418 for screening transformants of *C. sansevieriae*, and 100 µg/mL G418 for maintaining the transformants.

### DNA manipulation and plasmid construction

The genomic DNA of *C. sansevieriae* Sa-1-2 was extracted using a NucleoSpin Plant II kit (Takara Bio, Otsu, Japan) and used for further PCR amplification. The genomic information of the fungus was obtained from the NCBI database (NJHP000000002). The primers used in this work are all shown in Table S1.

First, to construct a selectable marker, the *NPTII* gene was amplified from pK18mob^38^ and the gene expression was controlled by the *gpdA* promoter and the *trpC* terminator amplified from pAN7-1^39^. The *NPTII* marker cassette was cloned into pBluescript II SK (+) (Agilent, Santa Clara, USA), yielding pBlue-G418. Next, to construct the gene replacement cassette to target the scytalone dehydratase gene (*SCD1*) involved in melanin biosynthesis, the upstream and downstream regions of *SCD1* were amplified and cloned into *Kpn*I and *Eco*RV sites of pBlue-G418, respectively, yielding pSCD1-G418 as a donor plasmid.

The endogenous U6 promoter in *C. sansevieriae* was predicted by identifying the U6 snRNA gene from the NCBI database^2^ of the fungus. Next, the 500-bp upstream promoter and 500-bp downstream terminator regions of the gene were amplified and then joined with single guide RNAs (sgRNAs) by PCR. Three target sequences in *SCD1* were designed using the webtool, CRISPRdirect (http://crispr.dbcls.jp): tg1, tg2, and tg3 (Fig. 3b). The *Cas9* gene with the SV40 NLS (PKKKRKV) was amplified from pFC332^30^, in which the gene was codon-optimized for translation in *A. niger* and under the control of the *tef1* promoter and terminator of *A. nidulans*. The Cas9-sgRNA expression cassettes were constructed by cloning the two fragments described above into *Eco*RV and *Spe*I sites of pBluescript II SK (+) (Agilent), respectively, yielding the three following plasmids: pCas9-sgRNA-tg1, -tg2, and -tg3. To construct a plasmid containing donor DNA-sgRNA cassettes for tg1, the sgRNA cassette was cloned into the *Spe*I site of pSCD1-G418, yielding pG418-sgRNA-tg1. In addition to *SCDI*, four other genes including two effector genes (*NIS1* and *MC69*), a cellulase gene (*Cel5A*), and an unknown gene (*g9136*) were also targeted, and plasmids containing each donor DNA-sgRNA cassettes for the four target genes were constructed in the same manner as described above using primers shown in Table S1.

All PCR products for cloning purposes were amplified using PrimeSTAR GXL Polymerase (Takara Bio) and cloned into plasmids via In-Fusion HD Cloning Kit (Takara Bio). For other purposes such as verification of replacement events, LA Taq DNA Polymerase (Takara Bio) was employed.

### *In vitro* assembly of a Cas9/sgRNA complex

To generate a sgRNA-encoding DNA, PCR was performed using pCas9-sgRNA-tg1 as a template, yielding a DNA template that contains the sgRNA sequence of tg1under the control of a T7 promoter. *In vitro*-transcribed sgRNAs were generated using a *CUGA*7 gRNA Synthesis Kit (Nippon Gene, Toyama, Japan), according to the manufacturer’s instructions. For the Cas9 cleavage assay, the transcribed sgRNAs and Cas9 NLS (Nippon Gene) were assembled and then mixed with PCR fragments containing its target site. After 1 h of incubation, the reaction mixture was subjected to agarose gel electrophoresis analysis.

### Transformation

The protoplasts of *C. sansevieriae* were generated from mycelial treatment with 1.2 M MgSO_4_ and 50 mM maleic acid (pH 5.5) containing 5 mg/mL Yatalase (Takara Bio) and 40 mg/mL VinoTaste Pro (Novozymes, Bagsværd, Denmark). Protoplasts were used at a concentration of 5 × 10^6^ or 1 × 10^8^ per transformation. Three micrograms of plasmid or PCR-amplified donor DNAs were introduced into protoplasts through PEG-mediated transformation^29^. For transformation by a Cas9/sgRNA complex, the complex was formed using 3 µg of Cas9 proteins and 1 µg of transcribed sgRNAs. To investigate whether Cas9 proteins can be transfected into protoplasts via PEG-mediated transformation, GenCrispr NLS-Cas9-EGFP (GenScript, Piscataway, USA) was used prior to the introduction of the complex. For the hybrid transformation, a Cas9 protein and a donor DNA-sgRNA expression plasmid were simultaneously introduced into protoplasts by PEG-mediated transformation. G418-resistant transformants were selected as described above.

## Supplementary information

Supplementary file
