# Supplementary material for: Plasmid-based and -free methods using CRISPR/Cas9 system for replacement of targeted genes in Colletotrichum sansevieriae
Source: Sci Rep. 2019 Dec 12;9:18947. doi: 10.1038/s41598-019-55302-8 (PMC6908651; doi:10.1038/s41598-019-55302-8)
Supplement: Supplementary file 1 — Supplementary file [file 41598_2019_55302_MOESM1_ESM.pdf]

Supplementary Information

Plasmid-based and -free methods using CRISPR/Cas9 system for replacement of targeted gene in *Colletotrichum sansevieriae*

Masayuki Nakamura*, Yuta Okamura, Hisashi Iwai

Faculty of Agriculture, Kagoshima University, Kagoshima, Japan

* Corresponding author

E-mail: masa@agri.kagoshima-u.ac.jp

Table S1. Primers used in this study.

Construction of pSCD1-G418		
Primer name	Gene	Sequence (5′- 3′)
G418-inf-F	*NPTII*	cggtatcgat *AAGCTT* TGCCATTAACCTAGGTACAG
G418-inf-R	*NPTII*	attcgatatc *AAGCTT* CAAGAGCGGATTCCTCAGTC
SCD1-Pro-inf-KpnI-F	*SCD1* upstream	gggcgaattg *GGTACC* TGGTCGTTCGAACTGGGTAG
SCD1-Pro-inf-KpnI-R	*SCD1* upstream	gggggggccc *GGTACC* GAAGGTGATGTTGCCAGCAG
SCD1-Ter-inf-EV-F	*SCD1* downstream	cgataagctt *GATATC* GCTACAACATGCACTGGTAC
SCD1-Ter-inf-EV-R	*SCD1* downstream	gcaggaattc *GATATC* CTGATGGGACGTTCCGATTG
Construction of pCas9 using pFC332 as a template		
Cas9-inf-EV-F	*Cas9*	atcgataagctt *GATATC* CGAGACAGCAGAATCACCGC
Cas9-inf-EV-R	*Cas9*	ctgcaggaattc *GATATC* GTATTGGGATGAATTTTGTA
Construction of pCas9-sgRNA-tg1, -tg2, -tg3, and pG418-sgRNA-tg1		
U6 Pro1046-inf-SpeI	U6 snRNA promoter	cgggggatcc *ACTAGT* TGGGCCTACGCAAACATGCG
U6 Pro1046-SCD1-tg1-B	U6 snRNA promoter	TGGAGTCGTAGCTGTCAGCCGAAAGTCTGTGGTAAAAGTG
SCD1-tg1-gRNA-C	sgRNA (tg1)	GGCTGACAGCTACGACTCCAGTTTTAGAGCTAGAAATAGC
gRNA-U6Ter-D	sgRNA (tg1)	GCATCAAAAAGGAAAAAAAAGCACCGACTCGGTGCCACTT
U6 Ter1046-E	U6 snRNA terminator	AAGTGGCACCGAGTCGGTGCTTTTTTTTCCTTTTTGATGC
U6 Ter1046-inf-SpeI	U6 snRNA terminator	ccgctctaga *ACTAGT* GGACGAGTGCGGAAAAGGCG
U6 Pro1046-SCD1-tg2-B	U6 snRNA promoter	GGGCGATGCACTTGCGGAGACGAAAGTCTGTGGTAAAAGTG
SCD1-tg1-gRNA-C	sgRNA (tg2)	TCTCCGCAAGTGCATCGCCCGTTTTAGAGCTAGAAATAGCAAG TT
U6 Pro1046-tg3-B	U6 snRNA promoter	AGATCATGGCAATGAACTCCGAAAGTCTGTGGTAAAAGTG
SCD1-tg3-gRNA-C	sgRNA (tg3)	GGAGTTCATTGCCATGATCTGTTTTAGAGCTAGAAATAGC
Amplification of donor DNAs using pSCD1-G418 as a template		

SCD1-up-1500-F	*SCD1* upstream	TGGTCGTTCGAACTGGGTAG
SCD1-down-1500-F	*SCD1* downstream	CTGATGGGACGTTCCGATTG
SCD1-up-1000-F	*SCD1* upstream	AACACTTGCGTGACATCCTG
SCD1-down-1000-F	*SCD1* downstream	ATGGCAGGTCAAACTAACCC
SCD1-up-500-F	*SCD1* upstream	TCAGTCGCCGACGTAAGTTG
SCD1-down-500-R	*SCD1* downstream	CTTCGTCTGTAGAGACTGTC
SCD1-up-50-F	*SCD1* upstream	TAATACACGGCGCATTCCGA
SCD1-down-50-R	*SCD1* downstream	GGCGAACTTCCACACGCCGT
Construction of sgRNA-encoding DNAs using pCas9-sgRNA-tg1 as a template		
T7-gRNA-synthesis-F	sgRNA (tg1)	CTAATACGACTCACTATAGGGCTGACAGCTACGACTCCA
gRNA-synthesis-R	sgRNA (tg1)	AAAAGCACCGACTCGGTGCC
Confirmation for gene replacements of *SCD1*		
SCD1-F	*SCD1*	TCCTCTAAGGTGGCCATGAC
SCD1-R	*SCD1*	ACACAGCCTCGAAGTCGTAC
Confirmation for integration of *Cas9* and sgRNA		
Cas9-F	*Cas9*	GGGATTCTTCAGACCGTCAA
Cas9-R	*Cas9*	AGCCAGCCTTATCGAGTTCA
gRNA-F	sgRNA	GGGATTACGACAAGCTAGTC
gRNA-R	sgRNA	CAAGTTGATAACGGACTAGC
Replacement of *NIS1*		
CsNIS1-Pro-KpnI-F	*NIS1* upstream	gggcgaattg *GGTACC* AGATGGGAAGCACTTGCAAC
CsNIS1-Pro-KpnI-R	*NIS1* upstream	ggggggggccc *GGTACC* TATTGTGCAGTTGCTGTGAG
CsNIS1-Ter-EV-F	*NIS1* downstream	cgataagctt *GATATC* ACAATGTCACCGTCAATGTG
CsNIS1-Ter-EV-R	*NIS1*	gcaggaattc *GATATC* TGGGACGTAGTAAGTTTACG

	downstream	
U6 Pro-CsNIS1-tg1-B	U6 snRNA promoter	GGACGGACTGGACGTAGTTCGAAAGTCTGTGGTAAAAGTG
CsNIS1-tg1-gRNA-C	sgRNA (*NIS1*)	GAACTACGTCCAGTCCGTCCGTTTTAGAGCTAGAAATAGC
CsNIS1-F	*NIS1*	ACTAGATGAGACGAGCAAAC
CsNIS1-R	*NIS1*	CGCCATTCATATGCAGCATG
Replacement of *MC69*		
CsMC69-Pro-KpnI-F	*MC69* upstream	gggcgaattg *GGTACC* CAGCATCGATAGCAGCGTAG
CsNIS1-Pro-KpnI-R	*MC69* upstream	ggggggggccc *GGTACC* CATCGTCAGGAGAGCTAATG
CsMC69-Ter-EV-F	*MC69* downstream	cgataagctt *GATATC* CCAGGCCTTTGCGAAGATGA
CsMC69-Ter-EV-R	*MC69* downstream	gcaggaattc *GATATC* GAGATGGTGTGTGCGGTATG
U6 Pro-CsMC69-tg1-B	U6 snRNA promoter	ACCCAGGCGACTGCTTCTTCGAAAGTCTGTGGTAAAAGTG
CsMC69-tg1-gRNA-C	sgRNA (*MC69*)	GAAGAAGCAGTCGCCTGGGTGTTTTAGAGCTAGAAATAGC
CsMC69-F	*MC69*	GTCCTTGCAATGCCCTTGTC
CsMC69-R	*MC69*	TGACGTGGAAGTGTGAGAAC
Replacement of *Cel5A*		
Cel5A-Pro-KpnI-F	*Cel5A* upstream	gggcgaattg *GGTACC* CGTGGGAAATTCAAAGACCG
Cel5A-Pro-KpnI-R	*Cel5A* upstream	ggggggggccc *GGTACC* CCTTGAATGAGTGACGAGAG
Cel5A-Ter-EV-F	*Cel5A* downstream	cgataagctt *GATATC* CCAACGACAAGGTCATCTTC
Cel5A-Ter-EV-R	*Cel5A* downstream	gcaggaattc *GATATC* GACATTCACTCGCTGCAGAG
U6 Pro- Cel5A-tg1-B	U6 snRNA promoter	GTGTTGTTCAGAATGACTACGAAAGTCTGTGGTAAAAGTG
Cel5A-tg1-gRNA-C	sgRNA (*Cel5A*)	GTAGTCATTCTGAACAACACGTTTTAGAGCTAGAAATAGC
Cel5A-F	*Cel5A-F*	GACTGGAATGTCGAAGTAGG
Cel5A-R	*Cel5A-F*	CCAGATCCAGTTCTCAGCAC

Replacement of *g9136*		
g9136-Pro-KpnI-F	*g9136* upstream	gggcgaattg *GGTACC* TAATGTCTCTCCTGACGGAT
g9136-Pro-KpnI-R	*g9136* upstream	ggggggggccc *GGTACC* CGGTAGAGTATTCCGAAGAC
g9136-Ter-SmaI-F	*g9136* downstream	attcctgcag *CCCGGG* AAAGCTGACGCACATCTCAG
g9136-Ter-SmaI-R	*g9136* downstream	tagtggatcc *CCCGGG* CGATCGTCTGTACCTTACGC
U6 Pro-g9136-tg1-B	U6 snRNA promoter	TTGTCGGTGCTGCCATCGCCGAAAGTCTGTGGTAAAAGTG
g9136-tg1-gRNA-C	sgRNA (*g9136*)	GGCGATGGCAGCACCGACAAGTTTTAGAGCTAGAAATAGC
g9136-F	*g9136*	CGACAACGAGATCCTTAATG
g9136-R	*g9136*	GACGACGTCAACTTACTACC

Lowercase sequences are homologous to vector ends. Italicized sequences represent restriction enzyme sites.

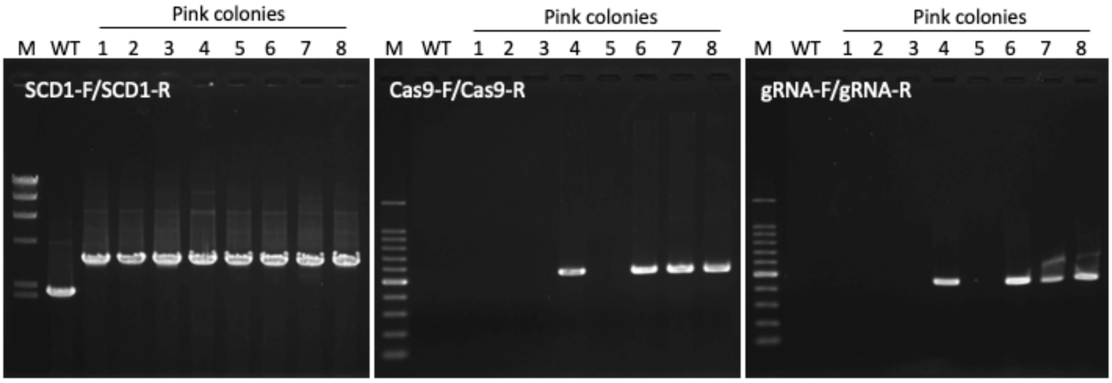

Figure S1. Full length gel images of the gels shown in Fig. 3d. Confirmation of *SCD1* replacement (left), Cas9-cassette integration (middle) and sgRNA-cassette integration (right) by PCR. Primers used are shown on the upper left. Gene replacement was caused by using pSCD1-G418 and pCas9-sgRNA-tg1 through the plasmid-based system.

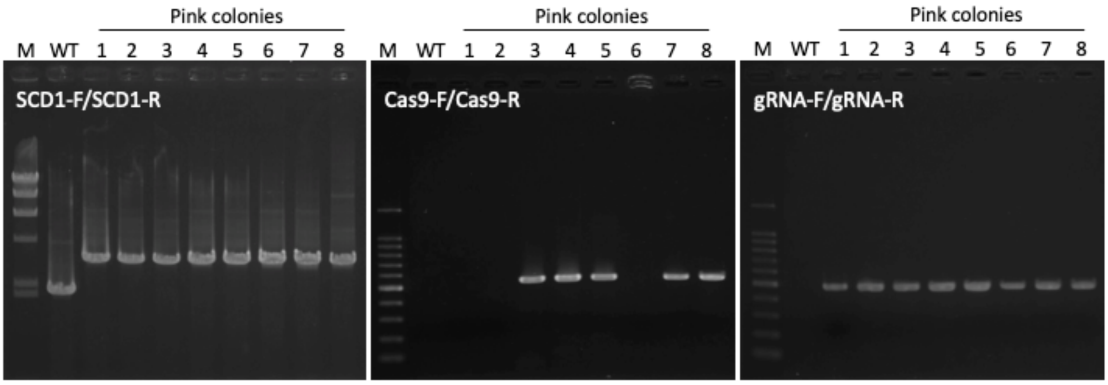

Figure S2. Full length gel images of the gels shown in Fig. 4c. Confirmation of *SCD1* replacement (left), Cas9-cassette integration (middle) and sgRNA-cassette integration (right) by PCR. Primers used are shown on the upper left. Gene replacement was caused by using pCas9 and pG418-sgRNA-tg1 through the plasmid-based system.

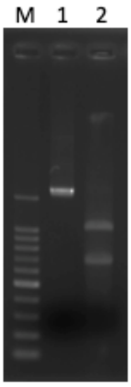

Figure S3. Full length gel image of the gel shown in Fig. 6a. Digestion of *SCD1* fragments without the Cas9/sgRNA complex (lane 1) or with the complex (lane 2).

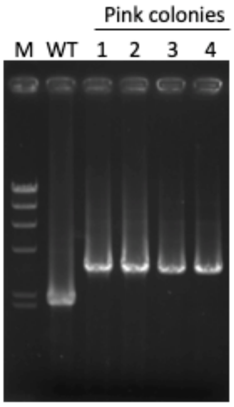

Figure S4. Full length gel image of the gel shown in Fig. 6d. Confirmation of *SCD1* replacement by PCR. Gene replacement was caused by the plasmid-free system (Cas9/sgRNA complexes).

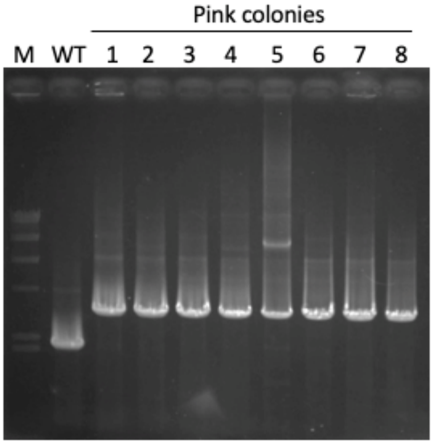

Figure S5. Full length gel image of the gel shown in Fig. 7c. Confirmation of *SCD1* replacement by PCR. Gene replacement was caused by the hybrid system (Cas9 protein and donor DNA-sgRNA expression plasmid).

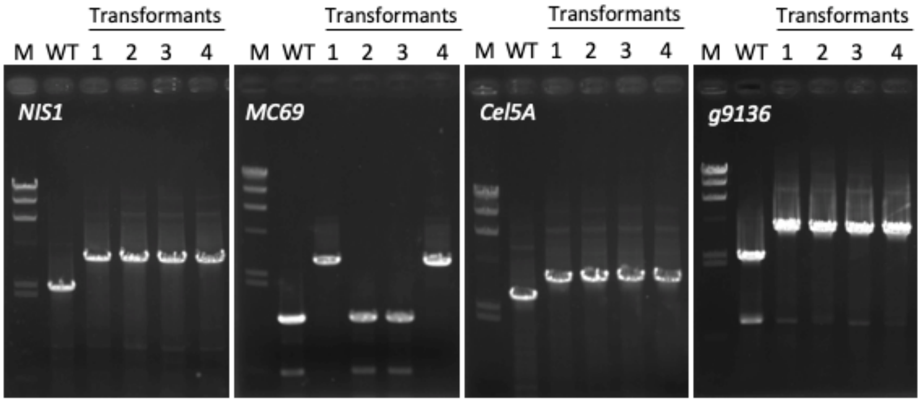

Figure S6. Full length gel images of the gels shown in Fig. 8. PCR analyses of replacement events for *NIS1, MC69, Cel5A,* and *g9136*. Gene replacements were caused by the hybrid system.
